# Association Between Maternal Physical Activity From Pre-pregnancy to Child-rearing and Their Children’s Physical Activity in Early Childhood Among Japanese

**DOI:** 10.2188/jea.JE20240041

**Published:** 2025-02-05

**Authors:** Aya Yamada, Haruki Momma, Nozomi Tatsuta, Kunihiko Nakai, Takahiro Arima, Chiharu Ota, Nobuo Yaegashi, Ryoichi Nagatomi

**Affiliations:** 1 Department of Medicine and Science in Sports and Exercise, Tohoku University Graduate School of Medicine, Miyagi, Japan; 2 Health and Environmental Risk Division, National Institute for Environmental Studies, Ibaraki, Japan; 3 The School of Sport and Health Science, Tokai Gakuen University, Aichi, Japan; 4 Department of Informative Genetics, Tohoku University Graduate School of Medicine, Miyagi, Japan; 5 Department of Development and Environmental Medicine, Tohoku University Graduate School of Medicine, Miyagi, Japan; 6 Department of Obstetrics and Gynecology, Tohoku University Graduate School of Medicine, Miyagi, Japan; 7 Division of Biomedical Engineering for Health and Welfare, Tohoku University Graduate School of Biomedical Engineering, Miyagi, Japan

**Keywords:** longitudinal, pregnant woman, preschool children, birth cohort

## Abstract

**Introduction:**

This study aimed to determine the association between cumulative maternal physical activity level and their children’s physical activity in early childhood. We also compared the influence of each maternal physical activity on children’s physical activity in early childhood.

**Methods:**

We analyzed the data from 1,067 Japanese mother-child pairs. Maternal physical activity was assessed using the International Physical Activity Questionnaire. Cumulative physical activity level in mothers was computed based on the categories (low, moderate, and high) of physical activity from five time points (pre-pregnancy, during pregnancy, 1.5, 3.5, and 5.5 years postpartum). Children’s physical activity level was measured at age 5.5 years using the WHO Health Behaviour School-aged Children questionnaire and defined as engaging in physical activity for at least 60 minutes per day for more than 5 days. Logistic regression analysis was used to determine the association between maternal and children’s physical activity levels.

**Results:**

The results showed the positive association between cumulative maternal physical activity and children’s physical activity level (*P* for trend < 0.001). Furthermore, maternal physical activity during pregnancy (*P* for trend = 0.031) and 5.5 years postpartum (*P* for trend < 0.001) was positively associated with children’s physical activity.

**Conclusion:**

A positive association was observed between the cumulative maternal physical activity level and the physical activity level of their children at 5.5 years of age. Furthermore, maternal physical activity during pregnancy and at 5.5 years postpartum were positively associated with the level of children’s physical activity.

## INTRODUCTION

Physical activity in childhood is beneficial for the prevention of childhood obesity and lifestyle-related diseases.^1^ Despite these benefits,^2^^,^^3^ 81.0% of children worldwide do not meet the World Health Organization (WHO)’s recommendation for physical activity.^4^ Since physical activity behaviors are established in early childhood and tracked into school age and adolescence,^5^^–^^7^ it is important that children acquire physically active behaviors from early childhood.

Parents can be thought of as mediators of children’s engagement in physical activity through direct or indirect approaches to their children. Direct approaches include playing with the child, sharing physical activity, encouraging play, providing the play opportunities, and parental support, while indirect approaches include acting as role models of physical activity and positive beliefs about physical activity.^8^^,^^9^ Indeed, a previous umbrella review reported that these parent-related factors and the parental physical activity itself are correlates of physical activity in early childhood,^8^ suggesting that parents and their behaviors greatly influence the physical activities of their children. Especially, mothers tend to spend more time with their children than fathers,^10^ so they are more likely to influence their children’s lifestyle behaviors. Previous cross-sectional studies showed that maternal, but not paternal, physical activity is positively associated with children’s physical activity.^11^^,^^12^ Given this, maternal physical activity may influence the acquisition of active behaviors in early childhood.

Cross-sectional studies showed positive associations between maternal physical activity and that of their children.^13^^–^^15^ However, longitudinal studies are limited.^11^^,^^16^^–^^20^ There are also limited studies that focused on early childhood.^17^^,^^19^^,^^20^ Although a previous study failed to detect the association of children’s physical activity in early childhood with maternal physical activity at 4 and 9 months postpartum,^17^ the other studies reported a positive association with maternal physical activity pre-pregnancy^20^ and at 2 years postpartum.^19^ Moreover, maternal physical activity during pregnancy^18^ and the child-rearing period^11^^,^^16^ is positively associated with children’s physical activity at school ages. These findings suggest that maternal physical activity at any point is fragmentally associated with children’s physical activity in early childhood. However, because maternal physical activity can change substantially during pregnancy and child-rearing,^21^ maternal physical activity levels from pre-pregnancy to the child-rearing period may not necessarily correlate and the positive association between maternal and child physical activity levels may not be consistently observed over time in the same population. Therefore, a more comprehensive and robust assessment of mothers’ physical activity over time, such as a cumulative exposure, is needed to capture the overall influence of maternal physical activity during these periods on children’s physical activity in early childhood. Cumulative physical activity reflects the total amount of physical activity over time and takes into account the change in physical activity by aggregating activity levels over time.^22^^,^^23^

The aim of study was to determine the influence of cumulative maternal physical activity during the pre-pregnancy to child-rearing period on children’s physical activity in early childhood in a Japanese population. In addition, because it is not clear at which time point during childhood maternal activity has the greatest influence on their children’s physical activity, we also compared the influence of each maternal physical activity on children’s physical activity in early childhood.

## METHODS

### Setting

This study was an Adjunct Study of the Japan Environment and Children’s Study (JECS) in Miyagi Prefecture. The JECS is a nationwide birth cohort study to determine environmental influences on children’s development and health.^24^ Between January 2011 to March 2014, 103,000 parent-child pairs were enrolled in the JECS, recruited from 15 regional unit centers including the Miyagi Regional Center (MRC) in Japan. The MRC registered 9,217 pregnant women and covers 14 municipalities in the Miyagi Prefecture, including coastal areas, such as Ishinomaki and Kesennuma. Of 9,217 pregnant women, a total of 3,793 parent-child pairs agreed to participate. This Adjunct Study was approved by the Ethics Committee of Tohoku University Graduate School of Medicine (approval no.; 2022-1-917). We obtained written informed consent from all the participants separately from the JECS.

### Study participants

The MRC obtained data from 3,826 respondents in the Adjunct Study. Individuals with more than two participation registrations (*n* = 439), multiple births (*n* = 58), foreign nationals (*n* = 2), missing data on maternal and children’s physical activity (*n* = 1,925), and confounding factors (*n* = 281) were excluded. Outliers in maternal physical activity (*n* = 53) were also excluded according to the International Physical Activity Questionnaire (IPAQ) analysis guidelines.^25^ Finally, 1,067 participants were included in the current study.

### Children’s physical activity

The Japanese version of the WHO Health Behaviour in School-aged Children (HBSC) questionnaire was used to assess moderate to vigorous physical activity (MVPA).^26^ In particular, the following question from the questionnaire was considered: “Over the past 7 days, on how many days were you physically active for at least 60 minutes?” Respondents answered with the number of days (0–7 days).^27^ This questionnaire was originally designed for self-reporting by school-aged children. As the child participants were preschool-aged in this study, the mothers provided the number of MVPA days on behalf of their children. This approach has not been validated but had to be used because of the need to have a consistent measure for comparison in our future follow-up study. We used a cut-off point of more or less than 5 days of at least 60 minutes of physical activity per day in line with previous studies.^28^

### Maternal physical activity

The IPAQ short form was used to assess maternal physical activity at 5 time points: pre-pregnancy, during pregnancy (second to third trimester), and 1.5, 3.5, and 5.5 years after the birth.^29^^–^^31^ Physical activity before pregnancy was recalled at the time of registration (first trimester). The IPAQ short form assessed walking, moderate-intensity physical activity, and vigorous-intensity physical activity. For each activity, mothers responded with the duration and frequency of the activity that lasted at least 10 minutes. Based on the IPAQ analysis guidelines,^25^ physical activity per week (measured by MET-minute/week) was computed and classified into three physical activity levels: low, moderate, and high. The criteria are provided in eMaterial 1. The physical activity categories were scored (low = 1, moderate = 2, high = 3), and the total score at 5 time points was calculated. Total physical activity scores were categorized in quartiles and considered as a main exposure. For secondary exposures, the IPAQ physical activity category (ie, low, moderate, and high physical activity) at each time point was used.

### Confounding factors

Potential confounding factors identified in previous studies included maternal age,^11^^,^^16^^,^^19^ education,^11^^,^^16^^–^^20^ body mass index (BMI; kg/m^2^),^11^ working status,^18^^,^^20^ pregnancy complication,^32^ household income,^11^^,^^19^ children’s sex,^11^^,^^16^^,^^18^^,^^20^ BMI z-score,^11^ number of siblings,^11^ and preschool attendance.^20^ Additionally, since sports experiences may influence subsequent physical activity,^33^ we considered maternal participation in sports club activities during high school as a confounding factor in this study. These variables were used from the time points that could be measured during the follow-up period. For variables measured at multiple time points, variables related to the mother were entered into the model at a point closer to the time of enrollment, and variables related to the children were entered into the model at a point closer to the outcome age of 5.5 years. The detailed measurement time points for each confounding factor are shown in eTable 1. Maternal education, sports experiences, working status, annual household income, children’s height and weight, number of siblings, and preschool attendance were obtained from a questionnaire completed by the mother. Maternal age, pregnancy complication, height, weight, and children’s sex were provided by the physician. Data on maternal weight and height before pregnancy (recalled at the time of registration) was used to calculate BMI. The lambda-mu-sigma (LMS) statistical method was used to calculate the children’s BMI z-score.^34^ Age- and sex-specific values of L, M, and S were derived using the Japanese growth curve criteria.^35^

### Statistical analysis

Participant characteristics are reported in quartiles of the maternal total physical activity score. The data are presented as medians and interquartile ranges for continuous variables, and as numbers and percentages for categorical variables. We used logistic regression analysis to calculate the odds ratios (ORs) and 95% confidence intervals (CIs) for children having 5 or more days of MVPA (treated as a binary outcome). The main independent variable was the maternal total physical activity score in quartiles. Model 1 was unadjusted, while in model 2, we adjusted for maternal age (continuous variable), BMI (continuous variable), pregnancy complication (without or with), education level (<13 years and ≥13 years), working status (not working or working), annual household income (<4 million Japanese yen, 4 to <6, and ≥6), children’s sex (girl or boy), BMI z score (continuous variable), child-care attendance (not attending or attending), and number of siblings (0, 1, and ≥2). In model 3, we additionally adjusted for participation in sports club activities during high school (no or yes). Furthermore, delayed childbearing (maternal age at delivery; <35 years and ≥35 years), having siblings, and children’s sex were examined in subgroup analyses.

As a secondary analysis, we investigated at which time point (pre-pregnancy, during pregnancy, at 1.5, 3.5, and 5.5 years postpartum) the influence of maternal physical activity was stronger. We performed a logistic regression analysis of the children’s physical activity level (≥5 days of MVPA) as the dependent variable and the maternal physical activity categories at each time point as the independent variable. Models 1, 2, and 3 were the same models as in the above analysis. In this analysis, an additional adjustment was made for physical activity at other time points as model 4. All analyses were performed using Stata 17.0 (StataCorp, LLC, College Station, TX, USA). A *P*-value less than 0.05 was considered statistically significant.

## RESULTS

**Figure 1.**
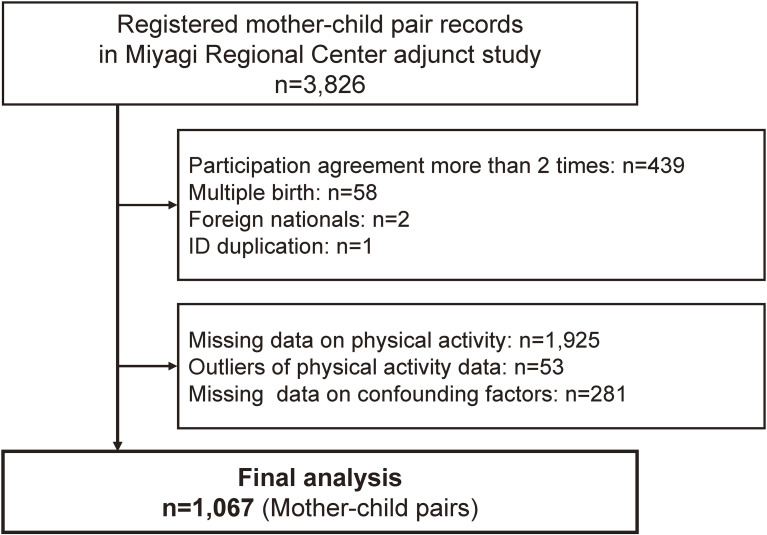
Flowchart of participants in this study

The study participant flowchart is shown in Figure 1. After excluding 2,759 cases, we analyzed 1,067 mother-child pairs. Participant characteristics in quartiles of cumulative maternal physical activity are shown in Table 1. Mothers with high cumulative physical activity levels during the pre-pregnancy to child-rearing period were characterized by previous sports club participation in high school. Table 2 shows information on maternal physical activity by quartiles of maternal total physical activity score. Mothers in quartile 1 (Q1) were not categorized in high physical activity at any time point. Moreover, the proportion of mothers meeting the Japanese^36^ and WHO^37^ guidelines for physical activity ranged from 23.2% (during pregnancy) to 38.3% (pre-pregnancy) and from 18.5% (during pregnancy) to 30.0% (pre-pregnancy), respectively. eTable 2 and eTable 3 provide more detailed information on maternal physical activity. Approximate 41.6% (pre-pregnancy) to 50.2% (5.5 years postpartum) of mothers in the “low” category reported 0 minutes/week of their physical activity at each time point. In addition, the mothers in Q1 have higher proportions of reporting 0 minutes/week at each time point (40.5% to 49.5%) as compared with the mothers in Q4 (1.2% to 8.7%) with a gradient reduction from Q1 to Q4.

**Table 1.** Participant characteristics

		Maternal physical activity total score			

	All (*n* = 1,067)	Q1 (*n* = 412)	Q2 (*n* = 154)	Q3 (*n* = 247)	Q4 (*n* = 254)
Maternal characteristics					
Age at delivery, year	31.0 (28.0–35.0)	32.0 (29.0–36.0)	31.0 (29.0–35.0)	31.0 (28.0–35.0)	30.0 (27.0–34.0)
BMI at pre-pregnancy, kg/m^2^	21.1 (19.5–23.3)	21.2 (19.5–23.4)	21.1 (19.7–23.0)	20.8 (19.2–22.5)	21.4 (19.7–23.8)
Pregnancy complications^a^					
Without	688 (64.5)	260 (63.1)	104 (67.5)	168 (68.0)	156 (61.4)
With	379 (35.5)	152 (36.9)	50 (32.5)	79 (32.0)	98 (38.6)
Education level, *n* (%)					
<13 years	447 (41.9)	185 (44.9)	62 (40.3)	83 (33.6)	117 (46.1)
≥13 years	620 (58.1)	227 (55.1)	92 (59.7)	164 (66.4)	137 (53.9)
Participation in sports club activities in high school, *n* (%)					
No	571 (53.5)	242 (58.7)	85 (55.2)	122 (49.4)	122 (48.0)
Yes	496 (46.5)	170 (41.3)	69 (44.8)	125 (50.6)	132 (52.0)
Working status at pre-pregnancy, *n* (%)					
Not working	323 (30.3)	123 (29.9)	47 (30.5)	87 (35.2)	66 (26.0)
Working	744 (69.7)	289 (70.2)	107 (69.5)	160 (64.8)	188 (74.0)
Annual household income during pregnancy, *n* (%)					
<4 million Japanese Yen	460 (43.1)	161 (39.1)	72 (46.5)	110 (44.5)	117 (46.1)
4 to <6	338 (31.7)	139 (33.7)	41 (26.6)	80 (32.4)	78 (30.7)
≥6	269 (25.2)	112 (27.2)	41 (26.6)	57 (23.1)	59 (23.2)

Child characteristics					
Sex, *n* (%)					
Girl	496 (46.5)	185 (44.9)	72 (46.8)	111 (44.9)	128 (50.4)
Boy	571 (53.5)	227 (55.1)	82 (53.3)	136 (55.1)	126 (49.6)
BMI z score at 4 years	0.34 (−0.26, 0.94)	0.34 (−0.29, 0.97)	0.19 (−0.31, 0.78)	0.31 (−0.19, 0.90)	0.43 (−0.23, 1.03)
Child-care attendance at 4 years, *n* (%)					
Not attending	142 (13.3)	51 (12.4)	30 (19.5)	33 (13.4)	28 (11.0)
Attending	925 (86.7)	361 (87.6)	124 (80.5)	214 (86.6)	226 (89.0)
Number of siblings at 3.5 years, *n* (%)					
0	301 (28.2)	127 (30.8)	48 (31.2)	66 (26.7)	60 (23.6)
1	508 (47.6)	189 (45.9)	65 (42.2)	123 (49.8)	131 (51.6)
≥2	258 (24.2)	96 (23.3)	41 (26.6)	58 (23.5)	63 (24.8)

BMI, body mass index.

Values are expressed as median (interquartile range) for continuous variables or *n* (%) for categorical variables, respectively.

^a^Pregnancy complication included cardiovascular disease (*n* = 3), threatened miscarriage (*n* = 139), threatened premature delivery (*n* = 216), premature rupture of the membranes (*n* = 58), placenta previa (*n* = 5), low-lying placenta (*n* = 1), cervical asthenia (*n* = 1), gestational hypertension (*n* = 32), preeclampsia (*n* = 1), vaginal bleeding (*n* = 4), and intrauterine growth restriction (*n* = 13).

**Table 2.** Maternal physical activity details

		Maternal total physical activity score			

	All (*n* = 1,067)	Q1 (*n* = 412)	Q2 (*n* = 154)	Q3 (*n* = 247)	Q4 (*n* = 254)
PA total score (range)	6–9	5–6	7	8–9	10–12
Total PA, MET-minutes/week					
Pre-pregnancy	693 (120–2,799)	130.8 (0–594)	495 (120–1,530)	1,200 (495–2,970)	3,600 (1,857–5,256)
During pregnancy	396 (0–1,188)	49.5 (0–347)	363 (0–792)	594 (198–1,188)	2,032.5 (655–4,158)
1.5 years postpartum	558 (0–1,980)	80 (0–378)	396 (0–1,032)	1,110 (480–2,586)	2,970 (1,200–4,878)
3.5 years postpartum	480 (0–1,733)	49.5 (0–396)	457 (0–1,137)	819 (240–1,950)	2,754 (996–4,410)
5.5 years postpartum	480 (0–1,695)	41.5 (0–376)	360 (0–990)	876 (240–1,943)	2,814 (946–4,320)
PA category, *n* (%)^a^					
Pre-pregnancy					
Low	550 (51.6)	357 (86.7)	94 (61.0)	78 (31.6)	21 (8.3)
Moderate	316 (29.6)	55 (13.4)	52 (33.8)	121 (49.0)	88 (34.7)
High	201 (18.8)	0 (0.0)	8 (5.2)	48 (19.4)	145 (57.1)
During pregnancy					
Low	703 (65.9)	385 (93.5)	106 (68.8)	148 (59.9)	64 (25.2)
Moderate	263 (24.7)	27 (6.6)	43 (27.9)	85 (34.4)	108 (42.5)
High	101 (9.5)	0 (0.0)	5 (3.3)	14 (5.7)	82 (32.3)
1.5 years postpartum					
Low	586 (54.9)	377 (91.5)	103 (66.9)	78 (31.6)	28 (11.0)
Moderate	323 (30.3)	35 (8.5)	43 (27.9)	137 (55.5)	108 (42.5)
High	158 (14.8)	0 (0.0)	8 (5.2)	32 (13.0)	118 (46.5)
3.5 years postpartum					
Low	618 (57.9)	371 (90.1)	96 (62.3)	107 (43.3)	44 (17.3)
Moderate	311 (29.2)	41 (10.0)	52 (33.8)	114 (46.2)	104 (40.9)
High	138 (12.9)	0 (0.0)	6 (3.9)	26 (10.5)	106 (41.7)
5.5 years postpartum					
Low	634 (59.4)	377 (91.5)	104 (67.5)	113 (45.8)	40 (15.8)
Moderate	271 (25.4)	35 (8.5)	36 (23.4)	103 (41.7)	97 (38.2)
High	162 (15.2)	0 (0.0)	14 (9.1)	31 (12.6)	117 (46.1)
≥23 MET-hours/week^b^					
Pre-pregnancy	409 (38.3)	39 (9.5)	44 (28.6)	118 (47.8)	208 (81.9)
During pregnancy	248 (23.2)	15 (3.6)	23 (14.9)	59 (23.9)	151 (59.5)
1.5 years postpartum	352 (33.0)	29 (7.0)	34 (22.1)	106 (42.9)	183 (72.1)
3.5 years postpartum	322 (30.2)	19 (4.6)	36 (23.4)	88 (35.6)	179 (70.5)
5.5 years postpartum	311 (29.2)	28 (6.8)	26 (16.9)	82 (33.2)	175 (68.9)
≥150 minutes/week^c^					
Pre-pregnancy	320 (30.0)	31 (7.5)	30 (19.5)	83 (33.6)	176 (69.3)
During pregnancy	197 (18.5)	21 (5.1)	18 (11.7)	40 (16.2)	118 (46.5)
1.5 years postpartum	253 (23.7)	17 (4.1)	22 (14.3)	70 (28.3)	144 (56.7)
3.5 years postpartum	237 (22.2)	19 (4.6)	25 (16.2)	53 (21.5)	140 (55.1)
5.5 years postpartum	261 (24.5)	33 (8.0)	21 (13.6)	67 (27.1)	140 (55.1)

MET, metabolic equivalent of task; PA, physical activity.

Values are expressed as median (interquartile range) for continuous variables or *n* (%) for categorical variables, respectively.

^a^IPAQ analysis guidelines and criteria.^25^ Details are given in eMaterial 1.

^b^Japanese physical activity guidelines.^36^

^c^WHO 2020 guidelines on physical activity and sedentary behaviour.^37^

The sociodemographic characteristics of mother-child pairs excluded from the study are presented in eTable 4. Interestingly, the proportion of individuals with more than 13 years of education was 13.3% higher among the study participants than those who were excluded. The physical activity details of mothers and children excluded from the study are presented in eTable 5. The median of maternal total physical activity during the pre-pregnancy and child-rearing period was higher in those excluded from the study than those included.

In Table 3, Spearman’s rank correlation coefficients between each measurement time point of maternal physical activity are presented. Correlation coefficients for the IPAQ categories are shown in Table 3A, and those for total physical activity in Table 3B. The correlation between the IPAQ categories and total physical activity was positive for all combinations of measurement time points (*P* < 0.001). For physical activity categories, the correlation coefficient between physical activity during pregnancy and at each child-rearing period was low (0.31 at 1.5 years postpartum, 0.21 at 3.5 years, and 0.25 at 5.5 years).

**Table 3.** The correlation of maternal physical activity at each time point

(**A**) Physical activity category					

	Pre-pregnancy	During pregnancy	1.5 years	3.5 years	5.5 years

Pre-pregnancy		0.40	0.37	0.32	0.28
During pregnancy	0.40		0.31	0.21	0.25
1.5 years postpartum	0.37	0.31		0.39	0.39
3.5 years postpartum	0.32	0.21	0.39		0.36
5.5 years postpartum	0.28	0.25	0.39	0.36	


(**B**) Total physical activity (MET-minutes/week)					

	Pre-pregnancy	During pregnancy	1.5 years	3.5 years	5.5 years

Pre-pregnancy		0.43	0.40	0.34	0.33
During pregnancy	0.43		0.37	0.32	0.30
1.5 years postpartum	0.40	0.37		0.45	0.42
3.5 years postpartum	0.34	0.32	0.45		0.44
5.5 years postpartum	0.33	0.30	0.42	0.44	


MET, metabolic equivalent of task.

Spearman’s rank correlation coefficient.

Table 4 shows the OR and 95% CIs for the prevalence of children’s MVPA for more than 5 days per week relative to the quartiles of cumulative maternal physical activity. The cumulative maternal physical activity level showed a positive association with children’s physical activity level (*P* for trend < 0.001). In model 3, the adjusted ORs for Q1 were 1.28 (95% CI, 0.58–2.83) in Q2, 1.58 (95% CI, 0.82–3.05) in Q3, and 3.72 (95% CI, 2.07–6.67) in Q4. The subgroup analyses based on maternal age at delivery (<35 years old vs ≥35 years) and children’s sex consistently showed a positive association, regardless of age or gender (eTable 6 and eTable 7). The analysis based on sibling status showed a positive association only among participants with siblings (eTable 8).

**Table 4.** Odds ratios of children’s physical activity level according to the maternal total physical activity score quartiles

	Maternal total physical activity score				

	Q1	Q2	Q3	Q4	*P* for trend
*n*	412	154	247	254	
Case, *n* (%)	20 (4.9)	10 (6.5)	19 (7.7)	38 (15.0)	
Model 1	Reference	1.36 (0.62–2.98)	1.63 (0.85–3.12)	3.45 (1.96–6.08)	<0.001
Model 2	Reference	1.28 (0.58–2.84)	1.59 (0.82–3.06)	3.74 (2.09–6.69)	<0.001
Model 3	Reference	1.28 (0.58–2.83)	1.58 (0.82–3.05)	3.72 (2.07–6.67)	<0.001

Values are presented as an odds ratio (95% confidence intervals).

Model 1 is crude model.

Model 2 is adjusted for maternal age (continuous variable), body mass index (continuous variable), pregnancy complication (without or with), education level (<13 years and ≥13 years), working status (not working or working), annual household income (<4 million Japanese Yen, 4 to <6, and ≥6), child’s sex (girl or boy), child’s body mass index z score (continuous variable), child-care attendance (not attending or attending), and number of siblings (0, 1, and ≥2). Model 3 is additionally adjusted for maternal participation in sports club activities in high school (no or yes).

In Table 5, the OR and 95% CIs for the incidence of children engaging in MVPA for more than 5 days per week are presented for each time point, based on the mother’s physical activity level. Maternal physical activity during pregnancy (*P* for trend = 0.031) and at 5.5 years postpartum (*P* for trend < 0.001) was positively associated with children’s physical activity. For maternal physical activity during pregnancy, the adjusted OR for mothers with moderate and high physical activity was 1.47 (95% CI, 0.85–2.57) and 2.24 (95% CI, 1.09–4.62), respectively, when compared to those with low activity levels. At 5.5 years postpartum, the OR was 1.21 (95% CI, 0.68–2.15) for mothers with moderate activity, and 2.38 (95% CI, 1.27–4.46) for those with high activity, using the low physical activity group as the reference. At 3.5 years postpartum, the association was not observed in model 3. No association was observed at pre-pregnancy and 1.5 years postpartum.

**Table 5.** Odds ratios of children’s physical activity level according to the maternal physical activity level at each time point

	Maternal physical activity category			
	Low	Moderate	High	*P* for trend
Pre-pregnancy, *n*	550	316	201	
Case, *n* (%)	39 (7.1)	29 (9.2)	19 (9.5)	
Model 1	Reference	1.32 (0.80–2.19)	1.37 (0.77–2.43)	0.222
Model 2	Reference	1.34 (0.81–2.23)	1.41 (0.78–2.53)	0.191
Model 3	Reference	1.34 (0.81–2.23)	1.40 (0.78–2.52)	0.197
Model 4	Reference	0.94 (0.55–1.63)	0.69 (0.34–1.39)	0.378

During pregnancy, *n*	703	263	101	
Case, *n* (%)	47 (6.7)	25 (9.5)	15 (14.9)	
Model 1	Reference	1.46 (0.88–2.44)	2.43 (1.31–4.54)	0.004
Model 2	Reference	1.61 (0.96–2.70)	2.66 (1.40–5.05)	0.002
Model 3	Reference	1.61 (0.96–2.70)	2.64 (1.39–5.01)	0.002
Model 4	Reference	1.47 (0.85–2.57)	2.24 (1.09–4.62)	0.031

1.5 years postpartum, *n*	586	323	158	
Case, *n* (%)	40 (6.8)	30 (9.3)	17 (10.8)	
Model 1	Reference	1.40 (0.85–2.29)	1.65 (0.91–2.99)	0.070
Model 2	Reference	1.40 (0.85–2.30)	1.73 (0.94–3.19)	0.058
Model 3	Reference	1.39 (0.84–2.30)	1.72 (0.93–3.18)	0.061
Model 4	Reference	0.99 (0.57–1.73)	0.98 (0.47–2.01)	0.870

3.5 years postpartum, *n*	618	311	138	
Case, *n* (%)	36 (5.8)	33 (10.6)	18 (13.0)	
Model 1	Reference	1.92 (1.17–3.14)	2.43 (1.33–4.41)	0.001
Model 2	Reference	1.79 (1.08–2.94)	2.50 (1.36–4.58)	0.001
Model 3	Reference	1.77 (1.07–2.93)	2.49 (1.36–4.57)	0.001
Model 4	Reference	1.53 (0.89–2.62)	1.66 (0.82–3.36)	0.093

5.5 years postpartum, *n*	634	271	162	
Case, *n* (%)	38 (6.0)	24 (8.9)	25 (15.4)	
Model 1	Reference	1.52 (0.90–2.59)	2.86 (1.67–4.90)	<0.001
Model 2	Reference	1.48 (0.86–2.54)	2.97 (1.71–5.15)	<0.001
Model 3	Reference	1.48 (0.86–2.54)	2.95 (1.70–5.14)	<0.001
Model 4	Reference	1.21 (0.68–2.15)	2.38 (1.27–4.46)	0.010

Values are presented as an odds ratio (95% confidence intervals).

Model 1 is a crude model.

Model 2 is adjusted for maternal age (continuous variable), body mass index (continuous variable), pregnancy complication (without or with), education level (<13 years and ≥13 years), working status (not working or working), annual household income (<4 million Japanese Yen, 4 to <6, and ≥6), child’s sex (girl or boy), body mass index z score (continuous variable), child-care attendance (not attending or attending), and number of siblings (0, 1, and ≥2).

Model 3 is additionally adjusted for participation in sports club activities in high school (no or yes).

Model 4 is additionally adjusted for physical activity at other time points.

## DISCUSSION

This study investigated the association of cumulative maternal physical activity during the pre-pregnancy and child-rearing periods with their children’s physical activity in early childhood. The results showed a positive association between the cumulative maternal physical activity level and their children’s physical activity level at 5.5 years old. We also examined the influence of maternal physical activity at each time point and found that only maternal physical activity during pregnancy and at 5.5 years postpartum showed a positive association with children’s physical activity level at 5.5 years old. Our findings indicate that overall maternal physical activity during the pre-pregnancy to child-rearing period positively impacts children’s physical activity in early childhood, although the association was not always consistent at each measured time point.

Previous studies investigating the association between maternal physical activity at various time points from preconception to the child-rearing period and children’s physical activity in childhood^11^^,^^16^^–^^20^ at different ages reported a positive association between maternal and children’s physical activity.^11^^,^^16^^,^^18^^–^^20^ However, some mothers’ physical activity can change dramatically from pre-pregnancy to the child-rearing period, and the patterns of change may be different among mothers.^21^ Consequently, the positive association between maternal and children’s physical activity may not be consistently observed from preconception to the child-rearing period in the same population. Therefore, it is important to capture the overall influence of maternal physical activity across the pre-pregnancy to child-rearing period. In this study, maternal physical activity was measured at five time points (pre-pregnancy, during pregnancy, 1.5, 3.5, and 5.5 years postpartum), and the cumulative physical activity during this period was evaluated. Our results showed that a higher cumulative physical activity level in mothers was associated with a higher level of their children’s physical activity at 5.5 years old. This result expands the findings from previous studies that have focused on maternal physical activity at each time.^11^^,^^16^^,^^18^^–^^20^

One possible factor contributing to a positive association between maternal and children’s physical activity is maternal experiences in exercising and physical activities. Among Japanese adult women, sports participation in high school was positively associated with physical activity in adulthood.^33^ For pregnant women, physical activity before pregnancy had a positive influence on physical activity during and after pregnancy.^21^^,^^38^ From these findings, past sports experiences and physical activity are characteristic of maternal physically active behaviors later in life. In our study, we adjusted for maternal sports participation in high school, which has not been considered in the previous studies,^11^^,^^16^^–^^20^ and confirmed a positive association between the cumulative maternal physical activity level and children’s physical. Considering this, maternal sports experiences only partially explained the positive association between the physical activity of mothers and their children.

Another potential mechanism is the mother’s support for the children’s physical activity. Trost et al proposed parental support, including providing transportation to places where children play sports and supervising their participation in sports, as a mediating factor in parent-child physical activity.^39^ In our study, it is possible, that mothers who were physically active during the pre-pregnancy to child-rearing period also had more opportunities to support their children’s physical activity at 5.5 years old. However, we did not measure parental support, and, therefore, the potential underlying mechanisms remain unclear.

Additionally, we also examined the association between maternal physical activity at each time point and children’s physical activity at 5.5 years of age. As expected, maternal physical activity at 5.5 years postpartum showed a positive association with children’s physical activity at 5.5 years old (ie, cross-sectional analysis), probably because of temporal proximity. This finding is consistent with the result reported by Jago and colleagues.^11^ They examined the association between maternal-children physical activity at 5–6 and 8–9 years of age and children’s physical activity at 8–9 years old but showed a positive association only at 8–9 years of age. Considering younger children have more opportunities to be transported and play with their mothers, it is possible that children with active mothers have.

Contrary to our expectations maternal physical activity at pre-pregnancy and 1.5 years and at 3.5 years postpartum did not positively associate with their children’s physical activity at 5.5 years old. This result suggests that the influence of maternal physical activity at a given time point is weakened when it is temporally distant from the children’s physical activity. In contrast, maternal physical activity level during pregnancy was positively associated with children’s physical activity. This result is consistent with the findings of Mattocks et al.^18^ In that study, mother’s leisure time activities during pregnancy were positively associated with the physical activity of their school-aged children. Our study confirmed a similar association in early childhood. Although Mattocks et al discussed that physical activity during pregnancy may be indicative of later maternal physical activity, and thereby contribute to the children’s physical activity, the correlation coefficient for physical activity during pregnancy with each child-rearing time point (1.5, 3.5, and 5.5 years postpartum) was low in this study (*r_s_* = 0.21–0.31). This result implies that physical activity during pregnancy might not necessarily be closely associated with later maternal physical activity, and other potential mechanisms, such as biological factors, may be involved.

This study has several limitations. The exclusion of participants from the analysis with even one missing physical activity or confounding factors may introduce unintended selection bias. When we compared the characteristics of the included sample (*n* = 1,067) with the excluded sample (*n* = 2,758), those excluded were mostly mothers with low educational levels and mothers and children with high physical activity. Considering this, the association between maternal and children’s physical activity may have been strengthened if the excluded mothers and children with high physical activity were included. Furthermore, considering that the participants in this study included residents of the coastal areas of Miyagi Prefecture, the impact of the Great East Japan Earthquake (March 2011) may have affected the maternal physical activity levels of certain participants in this study. However, the level of maternal physical activity in our study population was comparable to other studies.^21^^,^^40^^,^^41^

Second, the IPAQ short version was used to assess maternal physical activity in this study. This questionnaire may underestimate the maternal physical activity because it does not assess physical activity of less than 10 minutes. Conversely, since the questionnaire assesses total physical activity across different domains (leisure, home, occupation, and transfer), the associations observed in this study may be related to the total physical activity assessment of the participating mothers. In addition, the study used the HBSC questionnaire to measure children’s physical activity. This questionnaire was designed for school-aged children to self-report. However, as the children in this study were younger (preschool-aged) and we believed that it would be difficult for the children to answer the questionnaire directly, their mothers answered on their behalf. There is a lack of previous studies on the accuracy of the data and the reliability and validity of proxy reporting by parents when using the HBSC questionnaire for younger children. Considering that mothers have almost no chance to monitor their child’s physical activity while they are in the child-care center, mothers needed to speculate their child’s physical activity. Although it would have been desirable to sample some participants to assess children’s physical activity using objective devices, such as accelerometers, to check the reliability and validity of proxy reporting by parents, this was not achieved. This is one of the limitations of our study. Third, approximately half of mothers in the “low” category at each time point reported 0 minutes/week of their physical activity. A bias may exist if mothers with extremely low levels of physical activity tended to report low levels of physical activity in their children. Therefore, although our cumulative approach has some advantages, the influence of bias may also be cumulative when using this approach.

Finally, there are unmeasured confounders in this study, such as paternal factors.^19^^,^^42^^,^^43^ These factors may weaken the association between maternal and children’s physical activity.

### Conclusion

The cumulative maternal physical activity level showed a positive association with their children’s physical activity level at 5.5 years of age. Additionally, maternal physical activity during pregnancy and at 5.5 years postpartum were positively associated with their children’s physical activity level.

## SUPPLEMENTARY MATERIAL

eMaterial 1. Criteria for physical activity categories according to the IPAQ analysis guideline^25^

eTable 1. Measurement time points of confounding factors

eTable 2. Maternal physical activity details according to each category and time point

eTable 3. Proportion of mothers reporting 0 minutes/week according to maternal total physical activity score

eTable 4. Characteristics of the samples included and excluded in this study

eTable 5. Maternal and children’s physical activity details for samples included and excluded in this study

eTable 6. Odds ratios of children’s physical activity level according to the maternal total physical activity score quartiles by maternal age

eTable 7. Odds ratios of children’s physical activity level according to the maternal total physical activity score quartiles by child’s sex

eTable 8. Odds ratios of children’s physical activity level according to the maternal total physical activity score quartiles by having siblings
