# Supplementary material for: Association Between Maternal Physical Activity From Pre-pregnancy to Child-rearing and Their Children’s Physical Activity in Early Childhood Among Japanese
Source: J Epidemiol. 2025 Feb 5;35(2):81–9. doi: 10.2188/jea.JE20240041 (PMC11706679; doi:10.2188/jea.JE20240041)
Supplement: Supplementary file 1 [file je-35-081-s001.pdf]

eMaterial 1. Criteria for physical activity categories according to the IPAQ analysis guideline[25]

High physical activity

(a) Vigorous-intensity activity on at least 3 days achieving a minimum total physical activity of at least 1,500 MET-minutes/week.

OR

(b) Seven or more days of any combination of walking, moderate-intensity or vigorous-intensity activities achieving a minimum total physical activity of at least 3,000 MET-minutes/week.

Moderate physical activity

(a) Three or more days of vigorous-intensity activity of at least 20 minutes per day.

OR

(b) Five or more days of moderate-intensity activity and/or walking of at least 30 minutes per day.

OR

(c) Five or more days of any combination of walking, moderate-intensity or vigorous-intensity activities achieving a minimum total physical activity of at least 600 MET-minutes/week.

Low physical activity

Those individuals who not meet criteria for “high physical activity” or “moderate physical activity” are considered to have a “low physical activity”.

eTable 1. Measurement time points of confounding factors

		Time points											
		Pre	During	Birth	1 month	6 months	1 year	1.5 years	2 years	2.5 years	3 years	3.5 years	4 years
Mother	Age	○		●									
	Educational level		●										
	Participation in sports club activities in high school							●					
	BMI (Height and weight)	●				○				○			
	Working status	●	○				○					○	
	Annual household income		●								○		
Child	Sex			●									
	BMI z score (Height and weight)						○	○	○	○	○	○	●
	Number of siblings							○		○		●	
	Child-care attendance					○	○		○		○		●

●: Time points included in the statistical model, ○: Time points not included in the statistical model

Pre: pre-pregnancy, During: during pregnancy, X m: child is X months old, X y: child is X years old

Pre-pregnancy data were recalled and answered at the time of registration for participation in the JECS.

eTable 2. Maternal physical activity details according to each category and time point

	Maternal physical activity level at each time point		
Total PA, MET-minutes/week	Low	Moderate	High
Pre-pregnancy			
Participants[a]	550	316	201
Median	120	1485	4758
Interquartile range	0, 396	947, 2772	4095, 6570
Frequency of 0 minute/week[a]	229 (41.6)	0 (0)	0 (0)
During pregnancy			
Participants[a]	703	263	101
Median	99	1386	4548
Interquartile range	0, 396	693, 2586	4158, 6216
Frequency of 0 minute/week[a]	313 (44.5)	0 (0)	0 (0)
1.5 years postpartum			
Participants[a]	586	323	158
Median	99	1386	4619
Interquartile range	0, 360	834, 2340	4158, 6132
Frequency of 0 minute/week[a]	271 (46.3)	0 (0)	0 (0)
3.5 years postpartum			
Participants[a]	618	311	138
Median	49.5	1386	4692
Interquartile range	0, 360	798, 2133	4158, 5733
Frequency of 0 minute/week[a]	301 (48.7)	0 (0)	0 (0)
5.5 years postpartum			
Participants[a]	634	271	162
Median	0	1272	4452
Interquartile range	0, 339	756, 2079	3924, 5580
Frequency of 0 minute/week[a]	318, (50.2)	0 (0)	0 (0)

PA, physical activity.

[a] Values are expressed as n or n (%).

eTable 3. Proportion of mothers reporting 0 minutes/week according to maternal total physical activity score

	Maternal total physical activity score			
	Q1 (n=412)	Q2 (n=154)	Q3 (n=247)	Q4 (n=254)
PA total score (range)	5–6	7	8–9	10–12
Pre-pregnancy	167 (40.5)	35 (22.7)	24 (9.7)	3 (1.2)
During pregnancy	204 (49.5)	44 (28.6)	43 (17.4)	22 (8.7)
1.5 years postpartum	192 (46.6)	42 (27.3)	31 (12.6)	6 (2.4)
3.5 years postpartum	197 (47.8)	42 (27.3)	45 (18.2)	17 (6.7)
5.5 years postpartum	198 (48.1)	50 (32.5)	50 (20.2)	20 (7.9)

PA, physical activity.

Values are expressed as n (%).

eTable 4. Characteristics of the samples included and excluded in this study

	Included (n=1,067)	Excluded (n=2,758)
Maternal characteristics		
Age at delivery, years [a]	31.0 (28.0, 35.0)	30.0 (26.0, 34.0)
BMI at pre-pregnancy, kg/m^2 [b]	21.1 (19.5, 23.3)	21.1 (19.5, 23.6)
Pregnancy complications [c, d]		
Without	688 (64.5)	1,735 (64.7)
With	379 (35.5)	948 (35.3)
Education level, n (%) [e]		
<13 years	447 (41.9)	1,432 (55.2)
≥13 years	620 (58.1)	1,162 (44.8)
Participation in sports club activities in high school, n (%) [f]		
No	571 (53.5)	690 (56.2)
Yes	496 (46.5)	537 (43.8)
Working status at pre-pregnancy, n (%) [g]		
Not working	323 (30.3)	768 (29.4)
Working	744 (69.7)	1,848 (70.6)
Annual household income during pregnancy, n (%) [h]		
<4 million Japanese Yen	460 (43.1)	1053 (47.0)
4 to <6	338 (31.7)	682 (30.4)
≥6	269 (25.2)	507 (22.6)
Child characteristics		
Sex, n (%) [i]		
Girl	496 (46.5)	1,321 (49.4)
Boy	571 (53.5)	1,354 (50.6)
BMI z score at 4 years [j]	0.34 (-0.26, 0.94)	0.32 (-0.30, 0.93)
Child-care attendance at 4 years, n (%) [k]		
Not attending	142 (13.3)	184 (12.0)
Attending	925 (86.7)	1,346 (88.0)
Number of siblings at 3.5 years, n (%) [l]		
0	301 (28.2)	462 (25.3)
1	508 (47.6)	823 (45.0)
≥2	258 (24.2)	543 (29.7)

BMI, body mass index.

Values are expressed as median (interquartile range) for continuous variables or n (%) for categorical variables, respectively.

[a] n=2,708

[b] n=2,755

[c] n=2,683

[d] Pregnancy complication included cardiovascular disease, threatened miscarriage, threatened premature delivery, premature rupture of the membranes, placenta previa, low-lying placenta, cervical asthenia, gestational hypertension, preeclampsia, vaginal bleeding, and intrauterine growth restriction.

[e] n=2,594

[f] n=1,227

[g] n=2,616

[h] n=2,242

[i] n=2,675

[j] n=2,679

[k] n=1,530

[l] n=1,828

eTable 5. Maternal and children's physical activity details for samples included and excluded in this study

	Included (n=1,067)	Excluded (n=2,758)
Maternal total physical activity, MET-minutes/week		
Pre-pregnancy [a]	693 (120, 2,799)	777 (120, 2,970)
During pregnancy [b]	396 (0, 1,188)	396 (0, 1,485)
1.5 years postpartum [c]	558 (0, 1,980)	635 (80, 2,670)
3.5 years postpartum [d]	480 (0, 1,733)	539.5 (0, 2,167.5)
5.5 years postpartum [e]	480 (0, 1,695)	495 (0, 2,340)
Maternal physical activity category, n (%)[f]		
Pre-pregnancy [a]		
Low	550 (51.6)	1,336 (50.8)
Moderate	316 (29.6)	753 (28.6)
High	201 (18.8)	541 (20.6)
During pregnancy [b]		
Low	703 (65.9)	1,648 (63.8)
Moderate	263 (24.7)	638 (24.7)
High	101 (9.5)	298 (11.5)
1.5 years postpartum [c]		
Low	586 (54.9)	636 (52.2)
Moderate	323 (30.3)	342 (28.1)
High	158 (14.8)	240 (19.7)
3.5 years postpartum [d]		
Low	618 (57.9)	934 (57.1)
Moderate	311 (29.2)	433 (26.5)
High	138 (12.9)	269 (16.4)
5.5 years postpartum [e]		
Low	634 (59.4)	754 (58.6)
Moderate	271 (25.4)	321 (25.0)
High	162 (15.2)	211 (16.4)
Child's MVPA days [g]		
0	295 (27.7)	386 (30.1)
1	308 (28.9)	303 (23.6)
2	238 (22.3)	309 (24.1)
3	89 (8.3)	105 (8.2)
4	50 (4.7)	58 (4.5)
5	40 (3.8)	62 (4.8)
6	9 (0.8)	15 (1.2)
7	38 (3.6)	46 (3.6)

MVPA, moderate to vigorous physical activity.

Values are expressed as median (interquartile range) for continuous variables or n (%) for categorical variables, respectively.

[a] n=2,630

[b] n=2,584

[c] n=1,218

[d] n=1,636

[e] n=1,286

[g] n=1,284

[f] IPAQ analysis guidelines and criteria.[25] Details are given in eMaterial 1.

eTable 6. Odds ratios of children's physical activity level according to the maternal total physical activity score quartiles by maternal age

	Maternal physical activity total score				
	Q1	Q2	Q3	Q4	*P* for trend
<35 years					
N	272	107	180	195	
Case, n (%)	12 (4.4)	7 (6.5)	14 (7.8)	27 (13.9)	
Model 1	Reference	1.52 (0.58–3.96)	1.83 (0.82–4.05)	3.48 (1.72–7.06)	0.001
Model 2	Reference	1.37 (0.51–3.62)	1.63 (0.72–3.68)	3.52 (1.70–7.29)	0.001
Model 3	Reference	1.35 (0.51–3.58)	1.60 (0.71–3.61)	3.43 (1.65–7.11)	0.001
≥35 years					
N	140	47	67	59	
Case, n (%)	8 (5.7)	3 (6.4)	5 (7.5)	11 (18.6)	
Model 1	Reference	1.13 (0.29–4.43)	1.33 (0.42–4.23)	3.78 (1.43–9.96)	0.012
Model 2	Reference	1.16 (0.28–4.83)	1.12 (0.34–3.72)	4.07 (1.49–11.2)	0.016
Model 3	Reference	1.19 (0.28–5.00)	1.23 (0.37–4.14)	4.29 (1.55–11.9)	0.011

Values are presented as an odds ratio (95% confidence intervals).

Model 1 is unadjusted.

Model 2 is adjusted for maternal age (continuous variable), body mass index (continuous variable), pregnancy complication (without or with), education level (<13 years and ≥13 years), working status (not working or working), annual household income (<4 million Japanese Yen, 4 to <6, and ≥6), child's sex (girl or boy), body mass index z score (continuous variable), child-care attendance (not attending or attending), and number of siblings (0, 1, and ≥2).

Model 3 is additionally adjusted for maternal participation in sports club activities in high school (no or yes).

eTable 7. Odds ratios of children's physical activity level according to the maternal total physical activity score quartiles by child's sex

	Maternal physical activity total score				
	Q1	Q2	Q3	Q4	*P* for trend
Girl					
N	185	72	111	129	
Case, n (%)	10 (5.4)	5 (6.9)	6 (5.4)	17 (13.3)	
Model 1	Reference	1.31 (0.43–3.96)	1.00 (0.35–2.83)	2.68 (1.18–6.06)	0.029
Model 2	Reference	1.18 (0.37–3.69)	0.81 (0.28–2.34)	2.92 (1.26–6.76)	0.027
Model 3	Reference	1.17 (0.37–3.68)	0.82 (0.28–2.39)	2.96 (1.28–6.86)	0.024
Boy					
N	228	82	136	127	
Case, n (%)	10 (4.4)	5 (6.1)	13 (9.6)	21 (16.7)	
Model 1	Reference	1.41 (0.47–4.25)	2.29 (0.98–5.39)	4.34 (1.97–9.55)	<0.001
Model 2	Reference	1.44 (0.47–4.40)	2.36 (0.99–5.63)	4.69 (2.07–10.6)	<0.001
Model 3	Reference	1.42 (0.46–4.34)	2.29 (0.95–5.48)	4.57 (2.01–10.4)	<0.001

Values are presented as an odds ratio (95% confidence intervals).

Model 1 is crude model.

Model 2 is adjusted for maternal age (continuous variable), body mass index (continuous variable), pregnancy complication (without or with), education level (<13 years and ≥13 years), working status (not working or working), annual household income (<4 million Japanese Yen, 4 to <6, and ≥6), body mass index z score (continuous variable), child-care attendance (not attending or attending), and number of siblings (0, 1, and ≥2).

Model 3 is additionally adjusted for maternal participation in sports club activities in high school (no or yes).

eTable 8. Odds ratios of children's physical activity level according to the maternal total physical activity score quartiles by having siblings

	Maternal physical activity total score				
	Q1	Q2	Q3	Q4	*P* for trend
No siblings					
N	127	48	66	60	
Case, n (%)	8 (6.3)	2 (4.2)	7 (10.6)	7 (11.7)	
Model 1	Reference	0.65 (0.13–3.16)	1.76 (0.61–5.10)	1.96 (0.68–5.70)	0.140
Model 2	Reference	0.87 (0.17–4.44)	1.71 (0.55–5.27)	2.82 (0.91–8.75)	0.064
Model 3	Reference	0.87 (0.17–4.43)	1.72 (0.56–5.34)	2.85 (0.91–8.95)	0.066
Have siblings					
N	285	106	181	194	
Case, n (%)	12 (4.2)	8 (7.6)	12 (6.6)	31 (16.0)	
Model 1	Reference	1.86 (0.74–4.68)	1.62 (0.71–3.68)	4.33 (2.16–8.66)	<0.001
Model 2	Reference	1.57 (0.61–4.06)	1.35 (0.58–3.14)	4.58 (2.22–9.44)	<0.001
Model 3	Reference	1.56 (0.61–4.03)	1.34 (0.57–3.13)	4.57 (2.22–9.41)	<0.001

Values are presented as an odds ratio (95% confidence intervals).

Model 1 is crude model.

Model 2 is adjusted for maternal age (continuous variable), body mass index (continuous variable), pregnancy complication (without or with), education level (<13 years and ≥13 years), working status (not working or working), annual household income (<4 million Japanese Yen, 4 to <6, and ≥6), child's sex (girl or boy), body mass index z score (continuous variable), and child-care attendance (not attending or attending).

Model 3 is additionally adjusted for maternal participation in sports club activities in high school (no or yes).
